# Rehabilitating Compromised Dentition With All-on-6: A Multidisciplinary Clinical Approach

**DOI:** 10.7759/cureus.91610

**Published:** 2025-09-04

**Authors:** Chandrama Pratap, Radhika Sharma, Manisha Dahiya, Chingkheinganbi Nameirakpam, Rishabh Khare

**Affiliations:** 1 Prosthodontics, Swami Vivekanand Subharti University, Meerut, IND; 2 Prosthodontics, J. N. Kapoor D.A.V. Dental College, Yamunanagar, IND

**Keywords:** all-on-6 concept, direct metal laser sintering (dmls), implant-supported mouth recuperation, mandibular fixed dental prosthesis, maxillary fixed partial denture

## Abstract

This case report describes the rehabilitation of a patient using an implant‑supported fixed prosthesis in the mandible and a three‑unit fixed partial denture in the maxilla, following the extraction of periodontally compromised teeth. Six endosseous implants were placed in the mandibular arch, achieving favorable primary stability. The postoperative and maintenance phases were uneventful, with no biological or mechanical complications observed.

The maxillary arch was restored with a tooth‑supported fixed partial denture, planned to optimize both esthetics and function. At the four‑month follow‑up after delivery of the mandibular prosthesis, clinical evaluation confirmed the biological health of peri‑implant tissues, biomechanical integrity of the restoration, and proper occlusal function. The patient reported high satisfaction with both esthetic and functional outcomes.

For the definitive prostheses, direct metal laser sintering cobalt‑chromium frameworks veneered with ceramic were employed, owing to their advantages of precise marginal adaptation, favorable mechanical properties, and predictable long‑term performance.

## Introduction

Full-mouth rehabilitation is a comprehensive and challenging dental procedure that requires accurate diagnosis, systematic treatment planning, and precise clinical execution to restore oral function, esthetics, and patient comfort. Such cases are commonly associated with extensive dental caries, periodontal disease, or a combination of both, which often result in early tooth loss and impaired oral health.

Partial edentulism can be rehabilitated using various prosthetic modalities. While conventional removable or fixed prostheses are options, implant-supported restorations are considered the gold standard because of their superior retention, stability, esthetics, long-term predictability, and high patient satisfaction [1].

One modern treatment modality is the All-on-6 concept, which involves the strategic placement of six implants in an edentulous arch to support a full-arch fixed dental prosthesis. This technique maximizes available bone, often eliminates the need for bone augmentation, and is especially useful in cases with reduced bone density [2]. The All-on-6 approach offers optimal load distribution, biomechanical stability, and simplified clinical workflow [3].

For successful outcomes, rehabilitation requires not only predictable osseointegration but also fulfillment of esthetic and functional objectives, including proper soft tissue adaptation, emergence profile, occlusion, and prosthesis design [4]. Recently, direct metal laser sintering (DMLS) technology has become increasingly popular for fabricating implant-supported frameworks due to its digital precision, improved marginal adaptation, minimal porosity, and excellent mechanical properties, making it an ideal choice for customized fixed dental prostheses [5].

## Case presentation

Clinical report

A 38-year-old male patient presented to the Department of Prosthodontics with a chief complaint of poor esthetics and inability to chew due to multiple missing teeth. Comprehensive medical and dental histories were obtained, and full clinical and radiographic examinations were conducted. The patient had lost most mandibular teeth and several maxillary teeth, primarily as a result of chronic periodontitis and dental caries. Examination revealed the absence of teeth 14, 16, 24, and 26 in the maxillary arch, along with multiple missing teeth in the mandibular arch. The remaining teeth exhibited poor esthetic appearance and compromised periodontal condition.

Given the poor prognosis of the residual dentition and the patient’s expectations, a thorough treatment plan was formulated. Mandibular rehabilitation involved the extraction of the remaining teeth and placement of implant-supported fixed partial dentures. The maxillary arch was restored with fixed partial dentures divided into three segments: two three-unit fixed partial dentures (FPDs) for the posterior areas and one six-unit FPD for the anterior segment.

Two weeks after mandibular healing, border molding was performed for the edentulous ridge, followed by maxillomandibular relation recording and mounting on a semi-adjustable articulator. Radiographic assessment ensured the quality and quantity of available bone for implant placement. Treatment options, including tooth-supported and implant-supported prostheses, were explained to the patient. A provisional prosthesis was delivered during the healing period, and a silicone putty index was fabricated for the mandibular arch to guide the final restoration. Open-flap placement of Adin implants (4.5 × 11.5 mm) was performed in the regions of the canine, first premolar, and first molar.

As osseointegration progressed, rehabilitation of the maxillary arch was initiated. The silicone putty index guided occlusal reduction of abutments. Approximately 1.5 mm occlusal reduction, circumferential shoulder finish line, 1.5 mm axial reduction, and a total convergence angle of 6° were achieved. Polyvinyl siloxane was used for impressions, poured with Type IV die stone (Fuji Rock, GC). Marginal fit was verified, and final cementation of the maxillary FPDs was performed with Type I glass ionomer cement.

**Figure 1:**
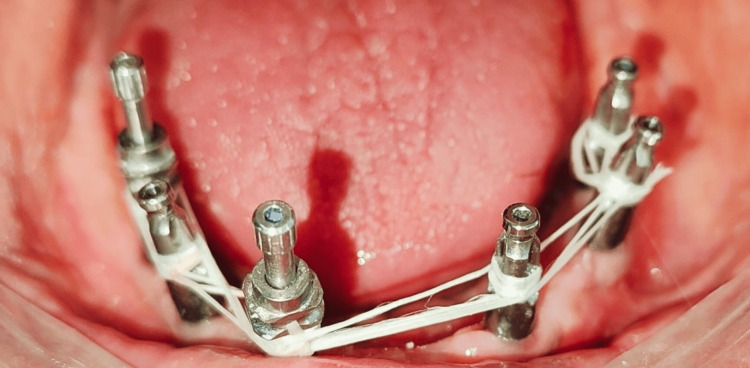
Intraoral splinting of impression coping using dental fluff Impression coping splinting is a dental technique used in implant dentistry to improve the accuracy of impressions, particularly for multiple implants.

**Figure 2:**
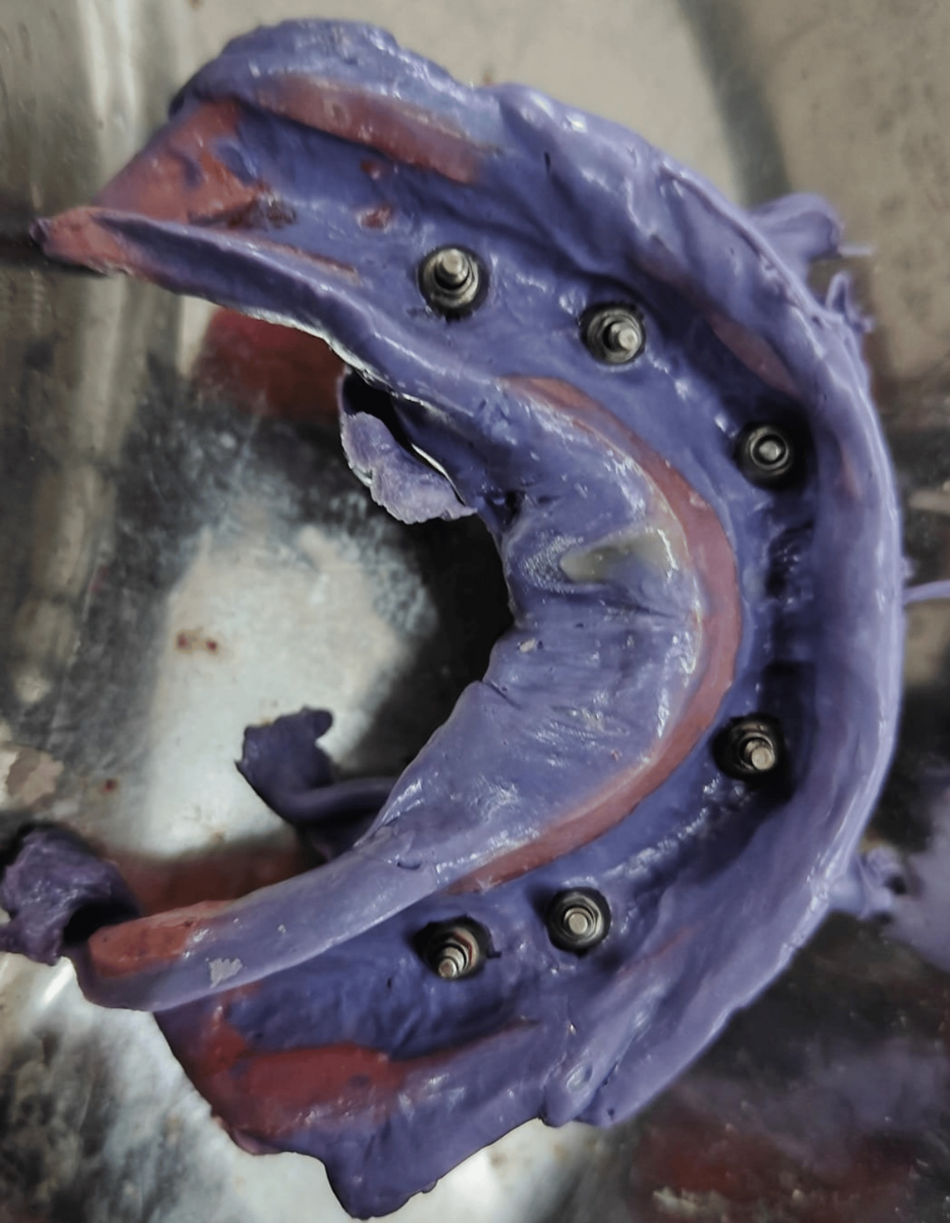
Final impression of dental implants An open customized tray was fabricated to make an imprint of a dental implant using PMMA.

**Figure 3:**
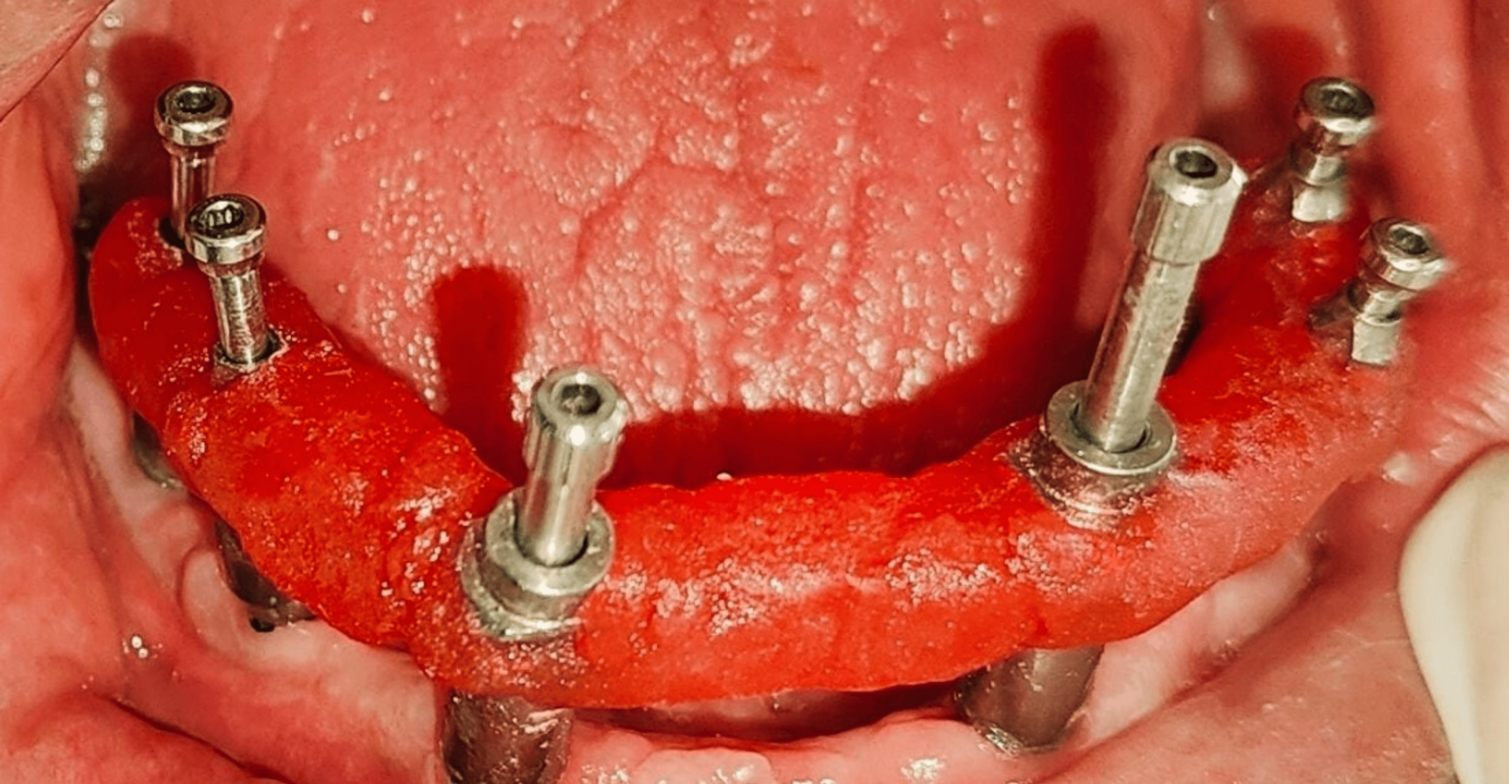
Intraoral jig trial An intraoral jig trial was performed to check the accuracy of the final imprint.

After four months, the second-stage surgery exposed the mandibular implants with the placement of healing abutments. Two weeks later, impression copings were splinted intraorally (Figure 1), and an open-tray impression was made using a custom tray of cold-cure resin (Figure 2). The verification jig confirmed the accuracy and stability of the impression (Figure 3).

**Figure 4:**
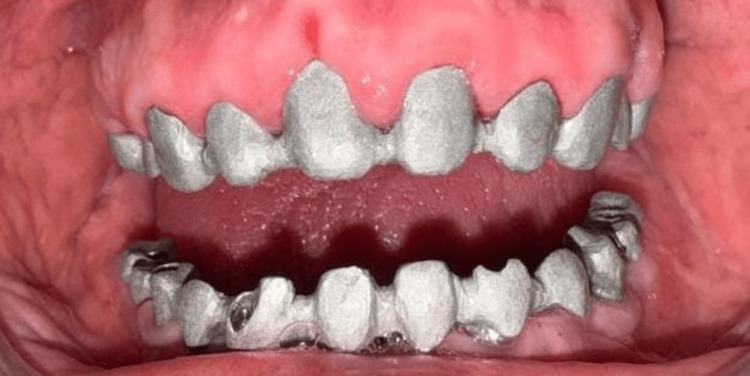
DMLS frame trial with respect to the lower arch and the three-unit metal trial with respect to the upper arch intraorally Direct metal laser sintering (DMLS) is a form of 3D printing. This process uses a laser to fuse metal powder into a precise, customized shape, often titanium. This material is known for its strength, durability, and good fit.

Metal frameworks were fabricated and tried in for precise fit and adaptation (Figure 4).

**Figure 5:**
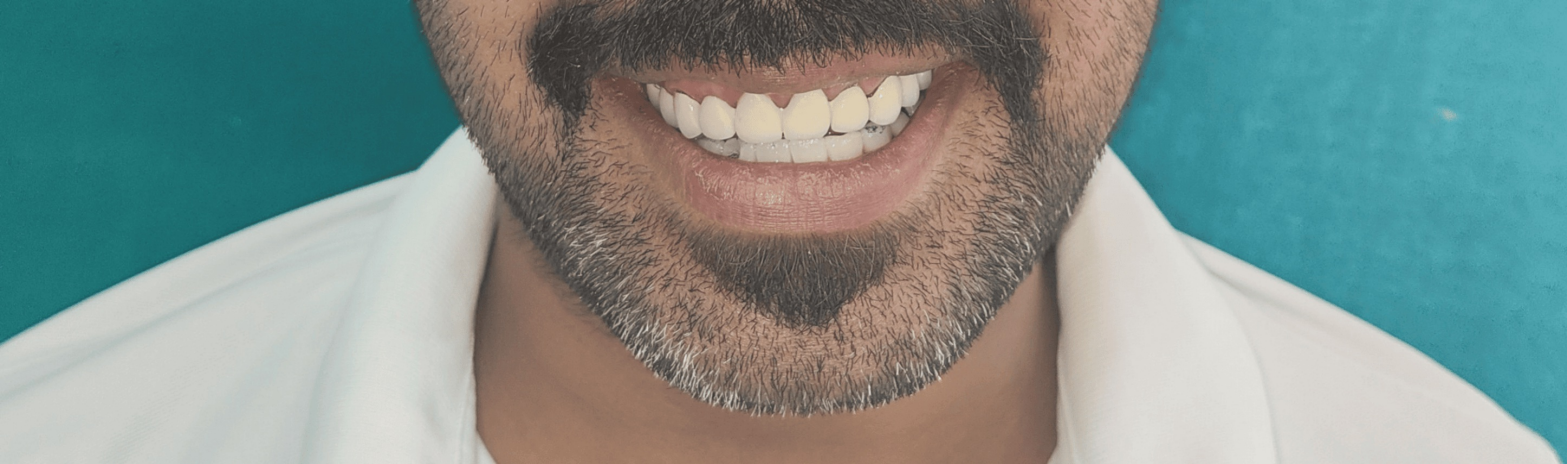
Final prosthesis The final mandibular prosthesis with ceramic veneering was delivered

The final mandibular prosthesis with ceramic veneering was delivered (Figure 5).

The patient was scheduled for follow-ups every six weeks for three months, then at six, 12, and 18 months post-insertion. Each visit included biological and mechanical assessment of the prostheses, confirming healthy peri-implant tissues and restoration stability throughout the follow-up period.

## Discussion

Accurate diagnosis and comprehensive treatment planning are essential for successful implant rehabilitation. Implant-supported FPDs are well-established as reliable, long-term solutions delivering predictable functional and esthetic outcomes. In this case, a segmented full-arch implant-supported prosthesis was selected for the mandibular arch to optimize occlusal load distribution, facilitate oral hygiene, and improve long-term prosthetic maintenance. Implant placement was guided by a prosthodontically driven approach to achieve ideal biomechanical alignment and optimal prosthetic positioning. A screw-retained prosthesis was used to ensure retrievability, allowing for ease of maintenance and management of potential complications such as screw loosening or restoration fracture [6].

Finite element analysis studies confirm that prostheses supported by five or six implants distribute occlusal forces more evenly than those supported by only three, thereby reducing the risk of mechanical overload and enhancing overall stability.

Advances in digital dentistry, particularly CAD/CAM technologies, have revolutionized prosthesis fabrication. DMLS, pioneered by Dr. Deckard and Beaman, fabricates metal frameworks layer by layer through laser sintering of fine metal powder [7]. DMLS restorations offer superior marginal adaptation and internal fit compared to conventional casting techniques, yielding significantly reduced marginal discrepancies (approximately 65 µm versus 125-150 µm for traditional methods).

For the maxillary arch, a conservative prosthodontic approach was adopted with porcelain-fused-to-metal crowns and FPDs, designed to satisfy the patient’s functional and esthetic requirements. The combined treatment strategy resulted in harmonious, functional, and esthetically pleasing full-arch rehabilitation.

## Conclusions

This case underscores the importance of precise diagnosis and multidisciplinary planning in complex full-mouth rehabilitation. The use of a segmented, implant-supported fixed prosthesis for the mandible-anchored by six endosseous implants-and a maxillary tooth-supported fixed partial denture provided predictable biomechanical stability, enhanced esthetics, and functional improvement for the patient. Integration of advanced digital techniques, especially DMLS for framework fabrication, enabled exceptional marginal adaptation and reinforced the long-term clinical success of the restorations. Regular follow-up revealed stable prosthetic and peri-implant outcomes without biological or mechanical complications.

Overall, comprehensive full-arch rehabilitation using modern implant and prosthodontic protocols can reliably restore oral function, esthetics, and patient satisfaction in individuals with extensive tooth loss due to periodontal disease and dental caries.
